# Dilation of Brain Veins and Perivascular Infiltration by Glioblastoma Cells in an *In Vivo* Assay of Early Tumor Angiogenesis

**DOI:** 10.1155/2021/8891045

**Published:** 2021-03-08

**Authors:** Quintino Giorgio D'Alessandris, Simone Pacioni, Vittorio Stumpo, Mariachiara Buccarelli, Liverana Lauretti, Martina Giordano, Rina Di Bonaventura, Maurizio Martini, Luigi M. Larocca, Stefano Giannetti, Nicola Montano, Maria Laura Falchetti, Lucia Ricci-Vitiani, Roberto Pallini

**Affiliations:** ^1^Institute of Neurosurgery, Department of Neuroscience, Fondazione Policlinico Universitario A. Gemelli IRCCS, Università Cattolica del Sacro Cuore, Rome 00168, Italy; ^2^Department of Oncology and Molecular Medicine, Istituto Superiore di Sanità, Rome 0061, Italy; ^3^Institute of Pathology, Fondazione Policlinico Universitario A. Gemelli IRCCS, Università Cattolica del Sacro Cuore, Rome 00168, Italy; ^4^Institute of Human Anatomy, Università Cattolica del Sacro Cuore, Rome 00168, Italy; ^5^CNR-IBBC, Institute of Biochemistry and Cell Biology, Consiglio Nazionale delle Ricerche, 00015 Rome, Italy

## Abstract

The cranial window (CW) technique provides a simple and low-cost method to assess tumor angiogenesis in the brain. The CW combined with histology using selective markers for tumor and endothelial cells can allow a sensitive monitoring of novel antiangiogenesis therapies in preclinical models. The CW was established in cyclosporine immunosuppressed rats that were stereotactically grafted with fluorescent U87MG glioblastoma cells. One to 3 weeks after grafting, brain vasculature was visualized *in vivo* and assessed by immunofluorescence microscopy using antibodies against endothelial and smooth-muscle cells and blood brain barrier. At 1-2 weeks after grafting, the CW reliably detected the hypertrophy of venous-venous anastomoses and cortical veins. These structures increased highly significantly their pregrafting diameter. Arterialized veins and hemorrhages were seen by three weeks after grafting. Immunofluorescence microscopy showed significant branching and dilation of microvessels, particularly those surrounded by tumor cells. Mechanistically, these changes lead to loss of vascular resistance, increased venous outflow, and opening of venous-venous anastomoses on the cortical surface. Data from the present study, namely, the hypertrophy of cortical venous-venous anastomoses, microvessel branching, and dilation of the microvessels surrounded by tumor cells, indicate the power of this *in vivo* model for the sensitive monitoring of early tumor angiogenesis.

## 1. Introduction

Glioblastoma (GBM) is a highly vascularized malignancy [1–4]. Studies on the early stages of angiogenesis as well as preclinical trials for antiangiogenic treatments require valuable *in vivo* models [5–8]. Xenografting onto the brain of immunocompromised rodents [9] recapitulates the interactions of human GBM with host endothelial cells [2] and extracellular matrix [10]. However, intracranial models need special tools to follow tumor evolution over time, such as high-field magnetic resonance imaging (MRI) [11, 12], microcomputed tomography [13, 14], or bioluminescence imaging [15, 16]. These techniques provide only indirect data on tumor angiogenesis and share disadvantages in terms of cost and technical expertise. In this report, we aim to validate the use of the cranial window (CW) technique for the direct visualization of the angiogenesis process in U87 brain tumor xenografts. Using this simple and low-cost technique, we were able to timely quantify the brain circulatory changes of tumor angiogenesis, in particular those involving the cortical venous-venous anastomoses, and to relate such changes with the histological picture.

## 2. Materials and Methods

### 2.1. Culture of Tumor Cells and Lentiviral Infection

The U87MG human GBM cell line was purchased from the American Type Culture Collection (Manassas, VA) [17]. Cells were cultured and virally transduced either for the green fluorescent protein (GFP) or for m-Cherry expression, as described elsewhere [18]. Cells were grown at 37° C in a humidified atmosphere of 5% CO2–95% air. Cells were regularly controlled to exclude mycoplasma contamination (Mycoalert Detection Kit, Lonza, Basel, Switzerland).

### 2.2. Intracranial Xenografting of Fluorescent U87MG Cells

Experiments involving animals were approved by the Institutional Ethical Committee (Pr. No. FF22). Study protocol was drawn in adherence with the International Association for the Study of Pain Guidelines for the Use of Animals in Research [19] and was fully compliant with the Directive 2010/63/EU on the protection of animals used for scientific purposes. Adult male Wistar rats (200–250 g; Catholic University Breeding Laboratory) were used. The rats were anesthetized with intraperitoneal injection of diazepam (2 mg/100 g) followed by intramuscular injection of ketamine (4 mg/100 g). For CW surgery, a 5 mm wide round craniectomy was made in the right fronto-parietal region under an operating microscope (Zeiss, Oberkochen, Germany). The dura mater was carefully opened. The craniectomy was covered with a round glass coverslip of 5 mm diameter and glued to the bone margins using cyanoacrylate. The skin was closed with metallic clips. Animals were kept under pathogen-free conditions and followed with daily measurements of weight, food and water consumption, and overall activity. Parenteral antibiotics were not given. Beginning 7 days before grafting, the rats were immunosuppressed with subcutaneous injection of cyclosporine (30 mg/kg, three times per week) [20]. Under general anesthesia, the animal skulls were immobilized in a stereotactic head frame and 2 × 10^5^ either of GFP+ or of m-Cherry+ U87MG cells were slowly injected using a 10 *μ*L-Hamilton microsyringe along a trajectory parallel to the cortex immediately below the pia mater via a hole made in the temporal bone. For *in vivo* brain imaging, the rats were anesthetized, body temperature was maintained at 38°C with a heating pad [21], and head fixed in the stereotaxic frame. The skin incision was reopened, and images of the CW were acquired at 10x magnification using the operating microscope equipped with a D5100 Nikon camera (Nikon Europe, Moncalieri, Italy). After 1-3 weeks survival, the rats were deeply anesthetized and transcardially perfused with 0.1 M PBS (pH 7.4) then treated with 4% paraformaldehyde in 0.1 M PBS. The brain was removed and stored in 30% sucrose buffer overnight at 4°C.

### 2.3. Fluorescence Microscopy and Immunofluorescence of Brain Tumor Xenografts

The brains were serially cryotomed at 40 *μ*m on the coronal plane. Sections were collected in distilled water and mounted on slides with Vectashield mounting medium (Bio-Optica, Milan, Italy). Images were acquired with a laser scanning confocal microscope (LSM 500 META, Zeiss, Milan, Italy). For immunofluorescence, sections were blocked in PB with 10% BSA, 0.3% Triton X-100 for 45 min and incubated overnight at 4°C with primary antibodies in PB with 0.3% Triton X-100 and 0.1% normal donkey serum (NDS). The monoclonal mouse anti-Glucose Transporter GLUT1 antibody (1 : 100; Abcam, Cambridge, UK) was used. Polyclonal antibodies used were as follows: rabbit anti-Glucose Transporter GLUT1 antibody (1 : 200; NovusBio, Centennial, CO, USA), rat antimouse CD31 (1 : 100) (BD Bioscience, Franklin Lakes, NJ), and goat anti-*α*-smooth muscle actin (*α*SMA) antibody (Abcam, Cambridge, UK). For detecting brain microvessels, sections were incubated overnight at 4°C in PB with 0.3% Triton X-100 and 0.1% NDS with Lectin from Lycopersicon esculentum (tomato) biotin conjugate (1 : 500; Sigma-Aldrich, St. Louis, MO) together with primary antibodies. Secondary antibodies used were as follows: Alexa Fluor 647 or 555 or 488 donkey antimouse, Alexa Fluor 488 or 555 or 647, donkey antirabbit secondary antibodies (1 : 500; Thermo Fisher Scientific, Waltham, MA), Alexa Fluor 488 or 555 donkey antigoat antibodies (1 : 400; Thermo Fisher Scientific, Waltham, MA), and Cy3 donkey antirat (1 : 200, EMD Millipore, Billerica, MA). For lectin immunostaining, sections were incubated for 2 h at room temperature in PB containing 0.3% Triton X-100 with streptavidin protein, DyLight 405 conjugate, or streptavidin Alexa Fluor® 647 conjugate (1 : 200; Thermo Fisher Scientific, Waltham, MA). To detect vascular permeability in brain xenografts, sections were incubated with Alexa Fluor 555 donkey anti-rat IgG (1 : 100; Abcam, Cambridge, UK) together with other primary antibodies. Before mounting, slices were incubated with DAPI (1 : 4000; Sigma-Aldrich) for 10 min.

As controls of fluorescence microscopy, we used rats (*n* = 9) treated for 1 to 3 weeks with subcutaneous injection of cyclosporine (30 mg/kg, three times per week). The specimens were observed with a laser confocal microscope (SP5; Leica), and images were acquired. Image analysis was performed with Leica Application Suite X software.

### 2.4. Statistical Analysis

Comparison of continuous variables at different time points during the study was performed using the paired Student's *t* test. Other comparisons of continuous variables were performed using the unpaired Student's *t* test. A *p* value less than 0.05 was considered statistically significant. Analyses were performed using the StatView v. 5.0 software (SAS Institute, Cary, NC).

## 3. Results

### 3.1. Cranial Window

**Figure 1:**
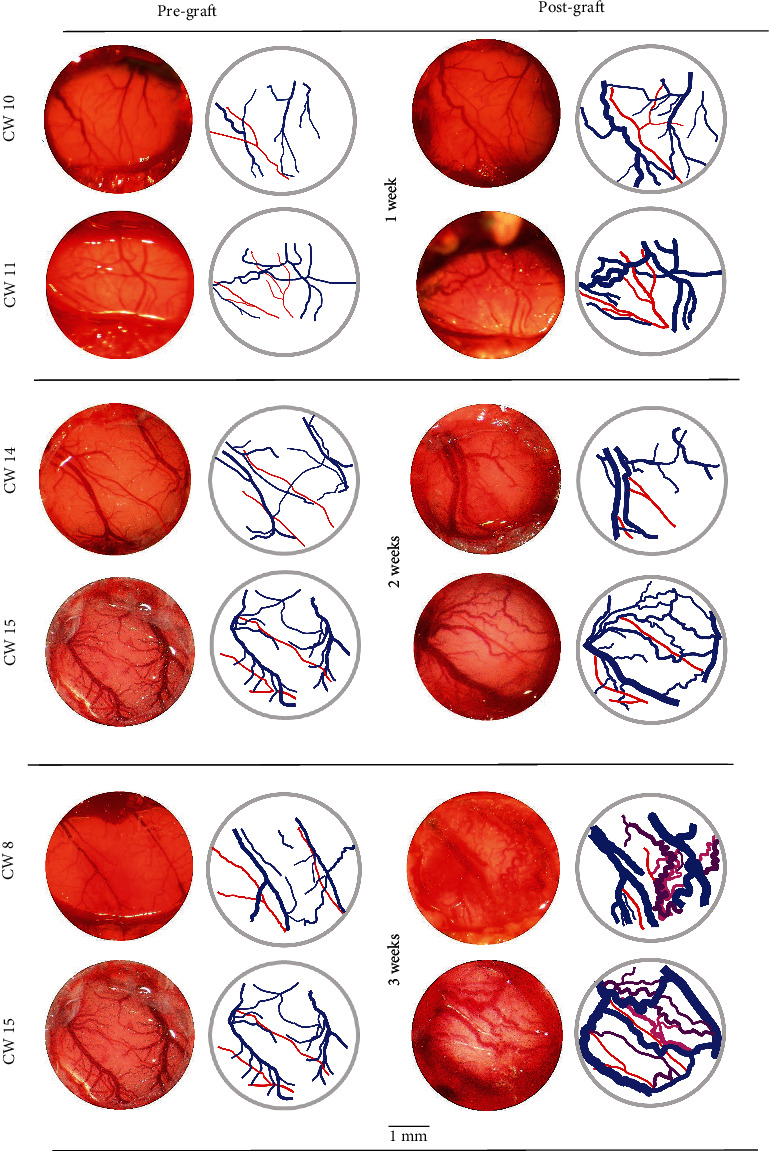
Microphotographs and computed image analysis of the CW performed before and 1 to 3 weeks after U87MG cell grafting. The first vascular change was hypertrophy of the venous-venous anastomoses due to opening of preexisting vessels. At 3 weeks after grafting, arterialized veins (*purple*) appeared on the cortical surface. Scale bar, 1 mm.

**Figure 2:**
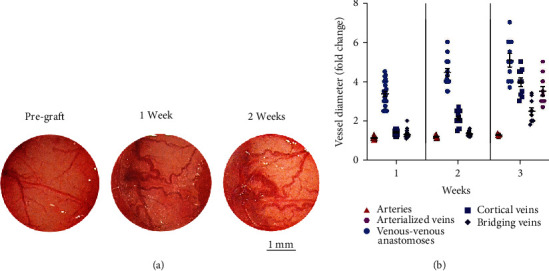
(a) Microphotographs of the CW performed before and 1 to 2 weeks after grafting of U87MG cells. Over this time frame, hypertrophy of the venous-venous anastomoses can be assessed without intervening hemorrhages that may obscure the phenomenon. (b) Graph showing the time course of diameter of arterial and venous vascular structures after grafting. One week after grafting, the diameter of venous-venous anastomoses and cortical veins increased significantly compared to their pregrafting diameter (*p* < 0.0001 and *p* < 0.001, respectively; paired Student's *t* test). Two weeks after grafting, the diameter of venous-venous anastomoses and cortical veins increased significantly compared to their pregrafting diameter (*p* < 0.001 and *p* < 0.0001, respectively; paired Student's *t* test). Three weeks after grafting, the vasculature was chaotic and heterogeneous with large-caliber vessels, arterialized veins, and hemorrhages.

The vascular changes were readily detected and observed over time by serial measurements after grafting (Figure 1). Upon surgical exposure, blood clots and scarring membranes were gently removed from the glass coverslip using gelfoam and saline irrigation at body temperature. In the first week after grafting, the main vascular change involved the cortical veins, particularly, the venous-venous anastomoses, which became hypertrophic (Figure 1). This phenomenon was not due to the formation of new vessels on the cortical surface but to the opening and hypertrophy of preexisting vessels. In the first week after grafting, the venous-venous anastomoses and cortical veins increased by 3.37 ± 0.13 (mean ± sem) and 1.41 ± 0.03 times their pregrafting diameter (*p* < 0.0001 and *p* < 0.001, respectively; paired Student's *t* test) (Figures 1 and 2). In this early phase, there were only minor and not significant changes in diameter of the middle cerebral artery branches and of the bridging veins to the superior sagittal sinus. At 2 weeks after grafting, the venous-venous anastomoses and cortical veins further increased their diameter by 4.46 ± 0.20 (mean ± sem) and 2.14 ± 0.10 fold their pregrafting diameter (*p* < 0.001 and *p* < 0.0001, respectively; paired Student's *t* test) (Figures 1 and 2). At this time, the arterial vessels still showed minor changes. At 3 weeks after grafting, dramatic changes occurred in the vascularity of brain cortex that included extreme dilation of the veins, appearance of tortuous and arterialized veins, and hemorrhages (Figure 1).

Overall, the first 2 weeks after grafting U87MG cells onto the brains of cyclosporine immunosuppressed rats represent the best time frame to detect the hypertrophy of venous-venous anastomoses and cortical veins without intervening hemorrhages, which may obscure the CW assessment (Figure 2). The tortuous arterialized veins were first seen by three weeks after grafting.

### 3.2. Fluorescence Microscopy of Brain Tumor Angiogenesis

For the histological assessment of tumor angiogenesis, we opted for lectin staining of the vascular structures. Both lectin and CD31 are regarded as selective markers of the vascular endothelium [10, 18, 22]. However, even in the brain of control rats, lectin stained a substantially higher number of microvascular structures than CD31 (Supplementary Figure S1), including the cordons of endothelial cells without central channeling. In control brains, the ratio between lectin-positive microvascular structures and CD31-positive microvessels was about 6-7 : 1.

**Figure 3:**
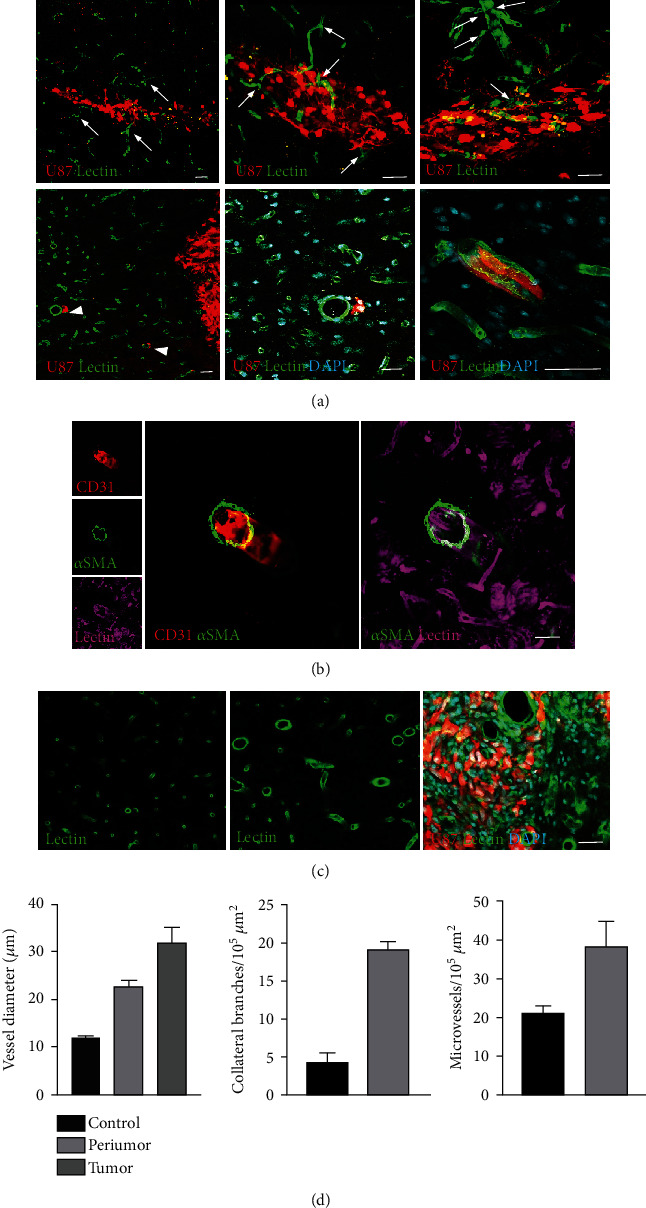
Fluorescence microscopy at 1 week after grafting mCherry+ U87MG cells onto the brain of cyclosporine immunosuppressed rats. (a) Collateral branching (*upper panel*, *arrows*) of microvessels contributing to the tumor (*red*). Dilated vessels surrounded by tumor cells (*lower panel, arrowheads*) that spread along perivascular spaces. Scale bars, 100 *μ*m. (b) Vessels with *α*SMA expression do not sprout. Scale bar, 25 *μ*m. (c) Progressive increase of microvessel diameter and density from control brain (*left*) to peritumor (center) and tumor (*right*) regions. Scale bar, 70 *μ*m. (d) Quantification of microvessel diameter, branching, and density.

At one week after grafting, the brain region within 500 microns from the grafting site (peritumor region) showed, (i) dilation of microvessels, particularly those surrounded by tumor cells, (ii) occurrence/increase of collateral branching by microvessels, and (iii) increase of the lectin-positive microvascular structures (Figure 3). In this early phase of tumor angiogenesis, one main vascular change involved the small vessels, capillaries, and postcapillary venules, into and around the tumor that appeared dilated and branched (Figure 3(a)). The vessels with *α*SMA-positive coverings, supposedly arterioles, contributed poorly to this early angiogenesis. The parent vessels, from which collaterals sprouted, were devoid of *α*SMA-positive coverings (Figure 3(b)). In the peritumor area, the dilated vessels were surrounded by tumor cells spreading along the perivascular space. Interestingly, the diameter of capillaries that were surrounded by tumor cells was significantly higher than that of the capillaries without perivascular tumor cells (*p* < 0.00001, Student's *t* test). In the capillaries, even as few as 2 tumor cells were capable to induce a 4-fold increase of the vessel diameter (Figure 3(a), lower panel). Both vessel dilation and branching could be readily assessed (Figure 3(d)). In the peritumor area and within the tumor, microvessels diameter was significantly greater than controls (*p* < 0.02 and *p* < 0.0001, respectively; Student's *t* test). In the peritumor region, branching was significantly higher than control brains (*p* < 0.00001, Student's *t* test; Figure 3(d)). The density of lectin positive microvascular structures was significantly higher than control brains (*p* < 0.0001, Student's *t* test; Figure 3(d)).

**Figure 4:**
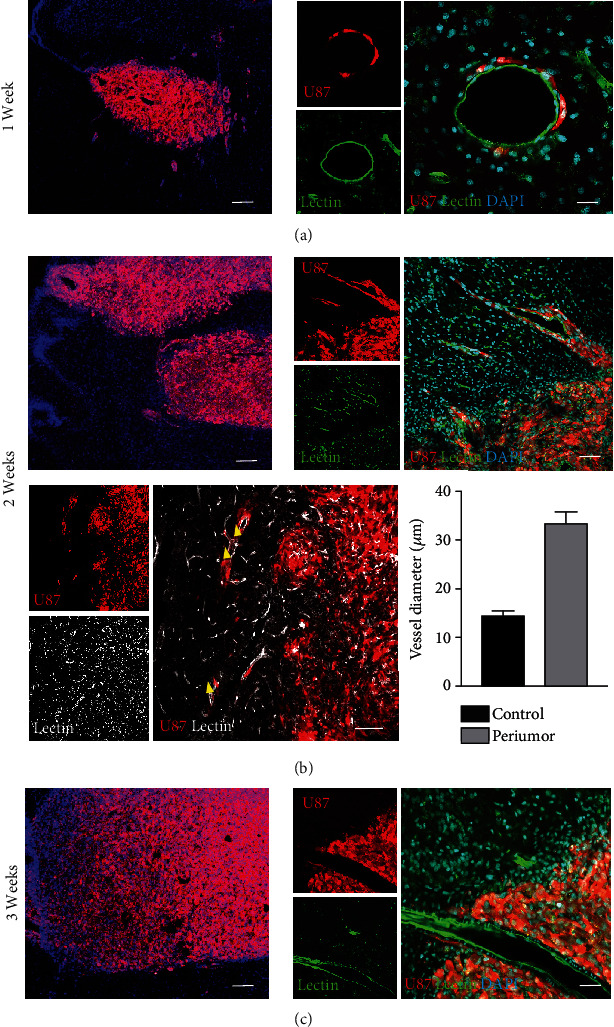
Fluorescence and immunofluorescence microscopy at 1, 2, and 3 weeks after grafting mCherry+ U87MG cells (*red*) onto the brain of cyclosporine immunosuppressed rats. (a) Microphotograph showing a tumor at 1 week after grafting (*left panel*). Dilation of vessels surrounded by tumor cells (*right panel*). Scale bars, 150 *μ*m (*left panel*) and 40 *μ*m (*right panel*). (b) The tumor at 2 weeks after grafting (*upper panel, left*). At the brain-tumor interface, dilated venules surrounded by cuffs of multilayered tumor cells (*upper panel, right*). Dilation of microvessels surrounded by tumor cells (*arrowheads*). The vessels within the tumor partly lose lectin staining (*lower panel, left*). Scale bars, 150 *μ*m (*upper panel, left*), 70 *μ*m (*upper panel, right*), and 70 *μ*m (*lower panel, left*). Graph showing the diameter of microvessels in controls and in peritumor regions (*lower panel, right*). (c) The tumor at 3 weeks after grafting (*left panel*). Extreme dilation and loss of lectin staining of microvessels within the tumor (*right panel*). Scale bars, 150 *μ*m (*left panel*) and 70 *μ*m (*right panel*).

By two weeks after grafting, the vessels within the tumor were enlarged and partly lost their lectin staining (Figures 4(a) and 4(b)). For these reasons, assessing the diameter of individual vessels within the tumor was not easy. At the brain-tumor interface, the venules became dilated at the point where their wall was surrounded by tumor cells, particularly when multilayered tumor cells arranged themselves to form cuffs (Figure 4(b)). Vessel dilation and branching could be assessed in the peri-tumor area (Figure 4).

**Figure 5:**
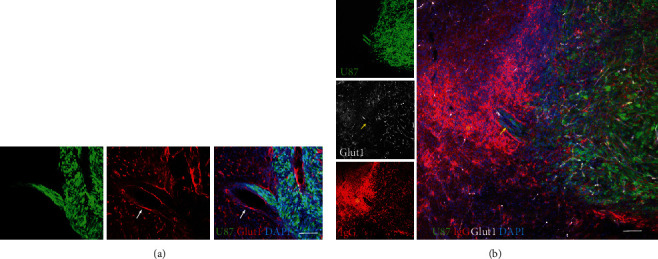
Immunofluorescence microscopy showing the expression of the BBB marker Glut1 and IgG extravasation in GFP-expressing U87MG brain xenografts. (a) At 2 weeks after grafting, the expression of Glut1 (*red*) by peritumor vessels is still present (*white arrow*). Scale bar, 70 *μ*m. (b) At 3 weeks after grafting, Glut1 expression (*white*) by vessels within and around the tumor is heavily disrupted (*yellow arrow*) with extravasation of IgG (*red*, *yellow star*). Scale bar, 100 *μ*m.

At 3 weeks after grafting, assessment of lectin-positive structures within the tumor did not provide reliable data given the extreme vessel dilation and partial loss of specific staining (Figure 4(c)). The vasculature within tumor showed chaotic and heterogeneous angioarchitecure with large-caliber vessels and discontinuous expression of lectin. One major change at this time was the disruption of BBB with IgG extravasation (Figure 5). The expression of Glut-1, a glucose transporter across the mammalian BBB [10], was highly decreased or lost in areas of reduced BBB integrity. Staining with anti-rat IgG highlighted extravasated immunoglobulins.

## 4. Discussion

The CW method [23] of U87MG brain xenografts in cyclosporine immunosuppressed rats offers a simple and rapid technique for assessing early angiogenesis of GBM, which correlates with tumor growth and vascular changes on histological analysis. By combining the CW technique with lectin fluorescence microscopy, we related the macroscopic vascular changes with remodeling of brain microvasculature. Novel findings of this study are the collateral branching of pre- and/or postcapillary microvessels and the dilation of microvessels surrounded by tumor cells. These phenomena lead to loss of vascular resistance, increased venous outflow, and opening of venous-venous anastomoses on the cortical surface.

The CW technique in U87 brain xenografts has been widely used to assess tumor angiogenesis under both light or fluorescence microscopy (Table 1) [24–46]. Major findings of these studies concerned the vessels within the tumor, which were larger than normal brain and showed increased permeability to serum borne proteins [24, 27, 35]. In the early phase (days 6-12 after grafting), the tumor vasculature was comprised mostly of preexisting brain capillaries undergoing vascular remodeling with enlargement, while new vessel formation occurred later [39]. Vascular remodelling with tortuous, dense, and swollen vessels showing decreased red blood cell velocity and rolling was also described [41, 43]. Then, the dilation of capillaries and postcapillary venules has long been recognized as an early event of tumor angiogenesis, which has been ascribed to the vascular endothelial growth factor (VEGF) and/or to other diffusible proangiogenic factors [47, 48].

In our study, microvascular dilation was strongly related to the spreading of tumor cells along the perivascular space. The tumor cells are able to travel along the perivascular spaces, where they cause dilation of the vessel wall [18, 49]. Previous studies that used clinically relevant models showed that glioma cells populate the perivascular space of preexisting vessels, displace astrocytic endfeet from endothelial or vascular smooth muscle cells, and disrupt the astrocyte-vascular coupling [49], a mechanism whereby the astrocytes regulate vascular tone through Ca(2+)-dependent release of K(+). Then, the dilation of microvessels may be due to the loss of vessel tone directly caused by perivascular tumor cells.

Other than the dilation of capillaries surrounded by tumor cells, another early event of GBM angiogenesis is the collateral branching of microvessels. An angiogenic sprouting was recognized as early as 3 days after C6 xenografts in nude mice [30, 32]. More recently, a highly branched vessel network was described to characterize the initial tumor growth of a mouse glioma model [45]. In the later stages, the branched pattern shifted to vessel expansion with loss of branching complexity. Vessel sprouts are supposed to arise from precapillary arterioles that contribute to the capillary network. In our study, however, the sprouting vessels have no *α*SMA coverings, suggesting that they may be aberrant arterioles or even postcapillary venules [22, 50]. In the latter instance, we would hypothesize a retrograde sprouting, whereby postcapillary venules contribute to the capillary network. We are well aware, however, that elucidating the mechanisms of vessel sprouting in early tumor angiogenesis warrants deeper insights. The extravasation of serum borne molecules and hemorrhages occurs at a late stage of tumor angiogenesis. Extravasation is clearly related to the disruption of the BBB, which does not occur in the early stages of angiogenesis [10].

The main limitation of this model relies on the cell line which has been chosen for the experiments, namely, the serum-cultured U87MG. U87MG cells xenotransplanted in immunocompromised rodents generate tumors which do not display an infiltrative growth [18] and tend to cause a wide disruption of the BBB [10]. Patient-derived glioma stem cells allow to build up a more clinically relevant GBM model [2, 9, 51]. On the other hand, U87MG is a highly angiogenic cell line endowed with the ability to grow rapidly [18], and it is thus very suitable for time-effectively assess GBM angiogenesis.

## 5. Conclusion

To conclude, the CW technique combined with histology using selective markers for tumor and endothelial cells can allow precise quantification of the venous-venous anastomoses on the brain cortex that are strongly linked to the dilation of the microvessels surrounded by tumor cells. These parameters, i.e., hypertrophy of venous-venous anastomoses, microvessel branching, and dilation of the microvessels surrounded tumor cells, can be readily assessed over two weeks after brain grafting. Our data suggest the power of this *in vivo* model for sensitive monitoring of novel antiangiogenesis therapies.

## Figures and Tables

**Table 1:** Literature review on angiogenesis in glioma models using the cranial window method.

Author, year	Model	Microscopy	Findings
Yuan, [24]	U87 and HGL21 xenograft, SCID mice	Intravital fluorescence microscopy	U87: vessel diameter 13.8 ± 2.4 *μ*m between days 12 and 18 HGL21: vessel diameter 6.8 ± 1.3 *μ*m. Vessel permeability high in U87, low in HGL21.
Foltz, [25]	Xenograft of human D-54 and D-317 anaplastic astrocytoma, nude rats	Light microscopy with epifluorescence	D-54 MG: vessel diameter 26.7 ± 12.0 *μ*m D-317 MG: vascular proliferation; vessel diameter 51.9 ± 11 *μ*m
Fukumura, [26]	HGL21 xenograft, SCID mice	Intravital fluorescence microscopy	Average vessel diameter 10 *μ*m at 2 weeks. No specific morphologic observations.
Yuan, [27]	U87 xenografts, SCID mice	Intravital fluorescence microscopy	Tumor vascular permeability 1.11 × 10^−7^ cm/s
Hobbs, [28]	U87 xenografts, SCID mice	Intravital fluorescence microscopy	U87: significant vasculature by 14-20 days
Monsky, [29]	HGL-21, SCID mice	Intravital fluorescence microscopy	Microvascular permeability to albumin ranging between 3.2 ± 0.9 and 6.4 ± 1.8 × 10^−8^ cm/s
Vajkoczy, [30]	C6 xenografts, nude mice	Intravital epifluorescence video microscopy and multiphoton laser confocal microscopy	Day 3: sprouting, chaotic and heterogeneous neovasculature with large-caliber vessels and sluggish blood flow vessel diameters: 12 *μ*m (day 3), 20 *μ*m (day 14). Blood-brain barrier: lost
Winkler, [31]	U87 xenografts, nude mice	Dynamic multiphoton laser scanning microscopy	Tumor vessel diameter about 1.5 *μ*m for 2-2.5 mm tumors.
Farhadi, [32]	C6 xenograft, nude mice	Intravital multifluorescence video microscopy	Day 3: microvascular sprouts from capillaries and venules, microvascular networks Day 10: vascularization of glioma by vessels with heterogeneous and chaotic angioarchitecture.
di Tomaso, [33]	U87 xenograft, nude mice	Multiphoton laser scanning microscopy	Day 15: vessels are larger than normal brain with diameter > 25 *μ*m
Winkler, [34]	GL261 graft, nude mice	Multiphoton laser scanning microscopy	Formation of capillary structures (glomeruloid bodies) in proximity to moving glioma cells
Kamoun, [35]	U87 xenografts, nude mice	Multiphoton laser scanning microscopy	Vessel permeability 1.5 − 2 × 10^−7^ cm/s, vessel diameter 14 *μ*m
Campos, [36]	GSC xenografts, SCID mice	Intravital fluorescence microscope	Neovascularization with dilated, tortuous capillaries in the tumor periphery.
Farrar, [37]	U87 xenograft, nude mice	Multiphoton laser-scanning microscopy	Vessel diameter of 9.5 ± 0.04 *μ*m for tumors with diameter 1.8 − 3.5 mm
Rege, [38]	9 L allograft rats	Laser speckle contrast imaging	Day 14 MVD values of 1.24 ± 0.13
von Baumgarten, [39]	U87, nude mice	Multiphoton laser scanning microscopy	Days 6-12: vascular remodeling with enlargement of preexisting brain capillaries Day 12: tumor diameter of 0.9-1.2 mm, new vessel formation diameter of 25 *μ*m.
Zhang, [40]	NA	In vivo two-photon imaging	No specific morphologic observations.
Ricard, [41]	U87, nude mice	Two-photon microscopy	Vascular remodeling, tumor vessels dense, tortuous and swollen, no correlation between tumor cell and vascular density
Ricard, [42]	GL261 graft, syngenic mice	Two-photon fluorescent microscopy	Vascular remodeling during tumor growth.
Takano, [43]	U87 xenografts, SCID mice	Fluorescence microscopy	Day 7: tortuous vessels with decreased velocity, leukocyte adhesion and rolling
Ricard, [44]	GL261 graft, syngenic mice	Two-photon microscopy	Vascular remodeling in tumor core
Mathivet, [45]	CT2A GL261 graft, C57Bl6 mouse	High-resolution two-photon microscopy	2 weeks: sprouting with normal caliber vessels and branching 5 weeks: reduced branching with increased vessel diameter Recruitment of M1-like macrophages in the early stages and M2-like macrophages producing VEGF-A in perivascular areas
Uhl, [46]	SF126 xenograft, nude mice	Intravital microscopy	Days 12-16: total vessel density 150 cm/cm^2^, functional vessel density 125 cm/cm^2^, vessel diameter 17 *μ*m

## Data Availability

Source data are available from the corresponding author upon reasonable request.

## Supplementary Materials

Supplementary Materials Supplementary Figure S1: Confocal microphotographs of brain microvessels in a control rat immunostained with endothelial markers. Lectin (*red*) stained much more microvessels compared to anti-CD31 staining (*green*). Branching of microvessels is rarely seen. Scale bar, 40 *μ*m.
