# Supplementary material for: Dilation of Brain Veins and Perivascular Infiltration by Glioblastoma Cells in an In Vivo Assay of Early Tumor Angiogenesis
Source: Biomed Res Int. 2021 Mar 8;2021:8891045. doi: 10.1155/2021/8891045 (PMC7960033; doi:10.1155/2021/8891045)
Supplement: Supplementary Materials — Supplementary Figure S1: Confocal microphotographs of brain microvessels in a control rat immunostained with endothelial markers. Lectin (red) stained much more microvessels compared to anti-CD31 staining (green). Branching of microvessels is rarely seen. Scale bar, 40 μm. [file 8891045.f1.pdf]

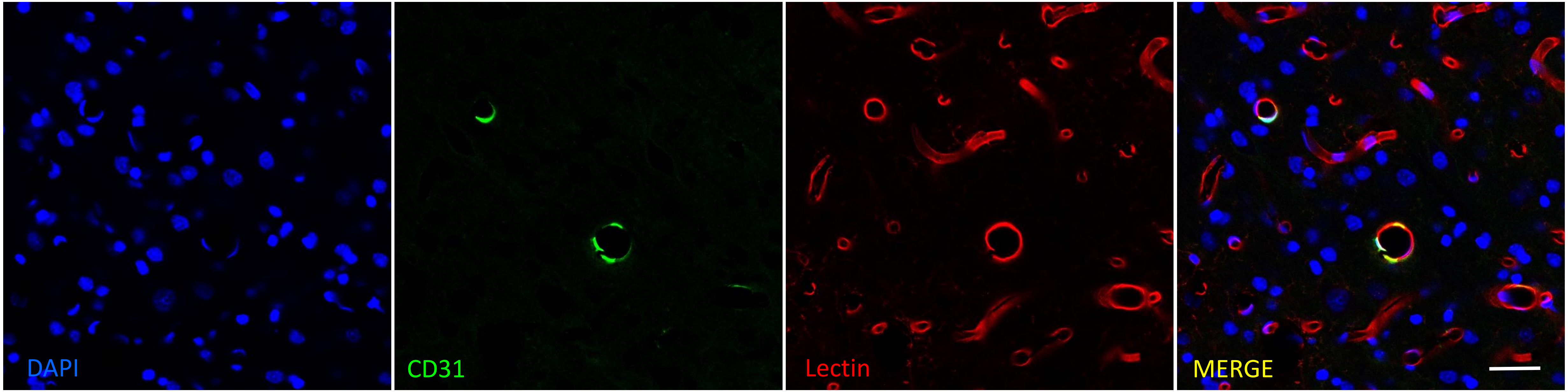

Supplementary Figure S1. Confocal microphotographs of brain microvessels in a control rat immunostained with endothelial markers. Lectin (*red*) stained much more microvessels compared to anti-CD31 staining (*green*). Branching of microvessels is rarely seen. Scale bar, 40 µm.
